# 6G and Artificial Intelligence Technologies for Dementia Care: Literature Review and Practical Analysis

**DOI:** 10.2196/30503

**Published:** 2022-04-27

**Authors:** Zhaohui Su, Barry L Bentley, Dean McDonnell, Junaid Ahmad, Jiguang He, Feng Shi, Kazuaki Takeuchi, Ali Cheshmehzangi, Claudimar Pereira da Veiga

**Affiliations:** 1 School of Public Health Southeast University Nanjing China; 2 Cardiff School of Technologies Cardiff Metropolitan University Cardiff United Kingdom; 3 Department of Humanities Institute of Technology Carlow Carlow Ireland; 4 Prime Institute of Public Health Peshawar Medical College Peshawar Pakistan; 5 Centre for Wireless Communications University of Oulu Oulu Finland; 6 Department of Research and Development Shanghai United Imaging Intelligence Shanghai China; 7 Ory Laboratory Inc Tokyo Japan; 8 Kanagawa Institute of Technology Kanagawa Japan; 9 Department of Architecture and Built Environment University of Nottingham Ningbo China Ningbo China; 10 Network for Education and Research on Peace and Sustainability Hiroshima University Hiroshima Japan; 11 Fundação Dom Cabral Nova Lima Brazil

**Keywords:** COVID-19, 6G, digital health, artificial intelligence, dementia, first-perspective health solutions

## Abstract

**Background:**

The dementia epidemic is progressing fast. As the world’s older population keeps skyrocketing, the traditional incompetent, time-consuming, and laborious interventions are becoming increasingly insufficient to address dementia patients’ health care needs. This is particularly true amid COVID-19. Instead, efficient, cost-effective, and technology-based strategies, such as sixth-generation communication solutions (6G) and artificial intelligence (AI)-empowered health solutions, might be the key to successfully managing the dementia epidemic until a cure becomes available. However, while 6G and AI technologies hold great promise, no research has examined how 6G and AI applications can effectively and efficiently address dementia patients’ health care needs and improve their quality of life.

**Objective:**

This study aims to investigate ways in which 6G and AI technologies could elevate dementia care to address this study gap.

**Methods:**

A literature review was conducted in databases such as PubMed, Scopus, and PsycINFO. The search focused on three themes: dementia, 6G, and AI technologies. The initial search was conducted on April 25, 2021, complemented by relevant articles identified via a follow-up search on November 11, 2021, and Google Scholar alerts.

**Results:**

The findings of the study were analyzed in terms of the interplay between people with dementia’s unique health challenges and the promising capabilities of health technologies, with in-depth and comprehensive analyses of advanced technology-based solutions that could address key dementia care needs, ranging from impairments in memory (eg, Egocentric Live 4D Perception), speech (eg, Project Relate), motor (eg, Avatar Robot Café), cognitive (eg, Affectiva), to social interactions (eg, social robots).

**Conclusions:**

To live is to grow old. Yet dementia is neither a proper way to live nor a natural aging process. By identifying advanced health solutions powered by 6G and AI opportunities, our study sheds light on the imperative of leveraging the potential of advanced technologies to elevate dementia patients’ will to live, enrich their daily activities, and help them engage in societies across shapes and forms.

## Introduction

The dementia epidemic is prevalent and pernicious [1], and it is engulfing the world at an alarming speed [2]. Approximately every 3 seconds, a new dementia case occurs, which translates into 10 million new annual cases added to the ever-increasing pool of dementia patients worldwide [3]. Globally, it is estimated that the number of people living with dementia will rise from 50 million in 2018 to 152 million in 2050 [2]. Dementia is not one disease but rather a family of health conditions that affect “memory and other cognitive abilities and behavior that interfere significantly with a person’s ability to maintain their activities of daily living” [4]. While Alzheimer’s disease is the most common type of dementia—accounts for 60% to 80% of all dementia cases, the term dementia could refer to a wide range of unique cognitive impairments rooted in diverse risk factors, ranging from Alzheimer’s disease, frontotemporal dementia, Lewy body dementia, vascular dementia, to mixed dementia [4]. While impacts such as adverse drug effects could also cause transitory or reversal dementia symptoms [5], unfortunately, due to limitations to developments in science and technology, the majority of dementia cases are difficult or impossible to reverse.

To further compound the matter, available pharmaceutical interventions that could effectively cure or curb dementia range from scarce to nonexistent, a situation which has been further plagued by controversies, rather than promoted by consensus, attracted by high-profile treatments such as Biogen’s aducanumab [6]. Overall, the increasing prevalence of dementia and the sobering lack of effective care could further aggravate the toll of the disease on lives, livelihoods, and economics. Take the more tangible economic consequences, for instance. It is estimated that the global economic cost of dementia has recently reached approximately US $1 trillion per annum [2] and is expected to inch towards US $9.12 trillion by 2050 [7]. What is too complex to materialize might be the most daunting task society has to shoulder—addressing the bevy of dementia needs and wants that are rooted in the kaleidoscopic range of symptoms that could manifest across the disease continuum [4].

The debilitating nature of the disease, particularly its corrosive impacts on patients’ cognitive and functional capabilities, dictates that dementia patients often have to depend on others for care and support [8]. Two main sources of care dementia patients rely on are professional health care providers (eg, doctors, nurses, and other formal caregivers) and informal caregivers (eg, family, friends, and other close social ties) [9]. Evidence suggests that, depending on contextual factors such as family values and cultural norms, family care could constitute from 65% to 96% of all health care services dementia patients receive [9-11]. While the existing laborious and caregiver-reliant care paradigm serves a purpose and holds well-deserved and hard-earned merits, it also hinders dementia care development—patients’ health and wellbeing, let alone access to care, are often contingent upon their informal caregivers’ could-be problematic abilities and ever-changing willingness to provide care [12-15].

Take caregivers’ dementia awareness and knowledge, for instance. In South Africa, for example, it is common for dementia patients, especially those of the female gender, to be considered as witches, even by health care professionals and patients’ family caregivers—as a result, rather than receiving quality care and pre-eminent support, these patients are often bullied, beaten, or burned many thanks to their caregivers’ exceedingly invalid but deeply ingrained dementia beliefs [13]. Furthermore, for some dementia caregivers, the seemingly never-ending, ever-worsening, and extremely-demanding duties and responsibilities associated with dementia care, particularly when compounded by (1) their lack of awareness, knowledge, and training, (2) competing interests and engagements, as well as (3) potential learned helplessness due to the continued absence of a tangible dementia cure, might be enough to trick or trigger them into committing inexcusable, yet possibly inescapable, neglect and abuse of dementia patients [16-18].

**Figure 1:**
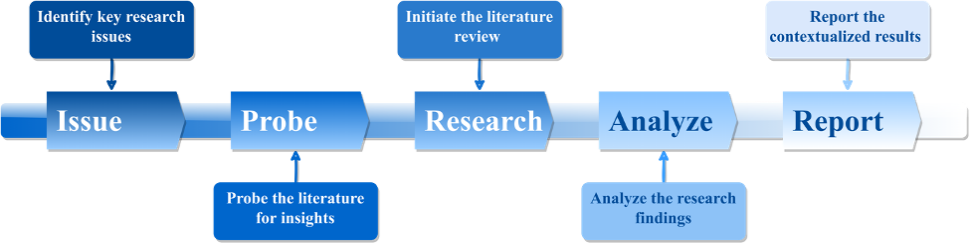
A schematic representation of the study framework.

These insights combined underscore the need for innovative approaches to address challenges patients, caregivers, health care professionals, as well as society at large face regarding dementia care. Overall, while an effective pharmaceutical solution to dementia or a magic bullet for fixing the patient-caregiver relationship might be difficult to secure, what can be relatively easy to change in order to improve the effectiveness and efficiency of dementia care and management is how current dementia services are designed and delivered [19-23]. In other words, the existing dementia caregiving model could possibly benefit from a paradigm shift. Rather than solely relying on laborious, time-consuming, and human-dependent solutions that suffer from great variability in terms of care quality [16-18]), efficient, cost-effective, and technology-based interventions could be the much-needed solution to tame the dementia epidemic, interventions such as those that are powered by advanced technological solutions such as the sixth-generation wireless technologies (6G) and artificial intelligence (AI) [24-26]. However, while 6G and AI technologies hold great promise, no research has examined how 6G and AI applications can effectively and efficiently address dementia patients’ health care needs and improve their quality of life. Thus, to bridge the research gap, this study aims to investigate ways in which 6G and AI technologies could elevate dementia care. A detailed research flow can be found in Figure 1.

## Methods

### Overview

To address the research objective, a literature review was conducted in databases such as PubMed, Scopus, and PsycINFO. The search was focused on three themes: dementia, 6G, and AI technologies. An example search string applied in PubMed could be found in Textbox 1. The initial search was conducted on April 25, 2021, focused on literature published in the past five years at the time. A subsequent search was conducted on November 21, 2021, to ensure all relevant insights were included in the review. Furthermore, Google Scholar Alerts focusing on dementia, 6G, and AI technologies were set up to make sure the review could incorporate the most recent evidence. In addition, we also included up-to-date, validated news reports in the review to ensure industry insights that are most relevant to the current study are included.

**Themes and search terms adopted.**

**Dementia:** dementia*[MeSH] OR dementia*[TIAB] OR Alzheimer*[MeSH] OR Alzheimer*[TIAB] OR “cognitive decline*”[MeSH] OR “cognitive decline*”[TIAB]

**AI:** “artificial intelligence”[MeSH] OR “artificial intelligence”[TIAB] OR “machine learning”[MeSH] OR “machine learning”[TIAB] OR “deep learning”[MeSH] OR “deep learning”[TIAB]

**6G:** “sixth-generation communication*”[MeSH] OR “sixth-generation communication*”[TIAB] OR “sixth-generation network*”[MeSH] OR “sixth-generation network*”[TIAB] OR “sixth-generation technolog*”[MeSH] OR “sixth-generation technolog*”[TIAB] OR “sixth-generation cellular” [MeSH] OR “sixth-generation cellular” [TIAB] OR “6G communication*”[MeSH] OR “6G communication*”[TIAB] OR “6G network*”[MeSH] OR “6G network*”[TIAB] OR “6G technolog*”[MeSH] OR “6G technolog*”[TIAB] OR “6G wireless”[MeSH] OR “6G wireless”[TIAB] OR “6G cellular”[MeSH] OR “6G cellular”[TIAB]

### Eligibility Criteria

The inclusion criteria adopted to screen relevant papers are listed in Textbox 2. Overall, we excluded articles if they (1) were not published in English, (2) did not focus on dementia patients (eg, dementia caregivers), (3) did not focus on either AI or 6G technologies, or (4) did not report empirical findings (eg, protocol studies), (5) did not focus on the noninvasive application of 6G or AI technologies (eg, collection of biomarkers such as blood), and (6) did not offer detailed information on the utilization of 6G or AI in the context of dementia care and management.

**Data type and study inclusion criteria.**

**Language:** English.

**Study context:** The use and application of 6G or AI in the context of dementia care and management.

**Technology type:** AI and 6G technologies.

**Use of technology:** Nonintrusive application of AI and 6G techniques or technologies.

**Study design:** Research that reports empirical findings.

### Review and Analysis Structure

Overall, as posited by the renowned social scientists Urie Bronfenbrenner [27], the quantity and quality of dementia care could be influenced by a wide array of factors, rooted in all levels of society—from the individual level, the interpersonal level, the community level, the social level, to the policy level. In light of the scope of the study, we only focused on dementia care accessed on the individual level and from the patients’ perspective. Furthermore, in order to comprehensively answer our overarching research aim—to understand ways in which 6G and AI technologies could elevate dementia care, two foundational research questions were identified and investigated: (1) what are the unique characteristics of dementia care, and (2) what 6G and AI-powered technologies could address the needs and wants of dementia patients. For ease of understanding and consistency in presentation, we organized the review and the subsequent analysis in accordance with these two research questions. A schematic representation of the review process can be found in Figure 1.

## Results

A total of 32 peer-reviewed articles were included in the final review. In addition, up-to-date and vetted news reports were examined to further enhance the rigor of the subsequent analysis. The overarching aim of this study is to investigate ways in which 6G and AI technologies could elevate dementia care. To effectively answer this research question, we first need to identify the unique characteristics of dementia care and then the specific 6G and AI-powered technologies that could address the logistics of dementia care. Subsequently, the results are divided into two sections, with the first one focusing on the unique characteristics of dementia care and the second section centering on specific ways in which 6G and AI technologies could be best leveraged to mitigate the health challenges dementia patients face. While complex in nature, drawing insights from the literature [28-34], dementia could be roughly categorized into three stages, the early, middle, and the late stage, each with unique disease manifestations, particularly in terms of patients’ cognitive and physical abilities (Table 1).

**Table 1:** Dementia care by stage.

Stage	Key characteristic	Care needed
Early stage	The symptoms are often overlooked due to the gradual onset of the disease	Regular forgetfulness Often lose track of time Frequently get lost in familiar places
Middle stage	The symptoms become clearer and more manifested over time.	Have difficulties in remembering recent events and acquittances’ names Confusion about time and space Incapable of communicating coherently Need assistance in self-care Behavioral irregularities like wandering and repeated questioning
Late stage	The symptoms become increasingly evident and debilitating.	Incognizance of time and space Unable to recognize relatives and friends Dependent on others for self-care Incapable to walk Behavioral irregularities like aggression and violence

Subsequently, the list of 6G and AI-powered technologies that could address the unique needs and preferences of people with dementia can be found in Table 2. In the following section, we will contextualize the findings of the study by delineating and dissecting the specific ways in which 6G and AI-powered technologies could be utilized to address people with dementia’s unique care needs and preferences.

**Table 2:** Key dementia care needs and advanced technology-based solutions

Dementia care needs and solution			Detail
**Memory impairment**			
	RFusion [35]	A robotic arm that could help dementia patients find even deeply hidden items based on camera and antenna data analyzed by advanced AI algorithms.	
	Egocentric Live 4D Perception [36]	An AI-powered project that could enable machines like virtual reality headsets to better help people like dementia patients to better navigate daily activities, ranging from finding lost items, limiting accidental over-medications, to enabling social interactions.	
Speech impairment	Project Relate [37]	An AI-powered communication tool built by Google that aims to help people with speech impairments communicate smoothly via Google Assistant.	
**Motor impairment**			
	Project Activate [38]	An AI-powered algorithm that allows people with speech and motor impairments to use facial expressions as smartphone commands.	
	Avatar Robot Café [39]	A robot and AI-powered system that allows people who are house-bound or bed-ridden, such as dementia patients, to engage in society as robot pilots—work remotely in the form of physical robot servers via virtually controlling these robots using an AI-powered system at home or even in bed.	
Cognitive and motor impairments	Affectiva [40]	An AI system that could recognize and analyze car drivers’ emotional and cognitive states, such as distraction, fatigue, and heatstroke, information which can then be used to send alerts to the drivers to prevent potential accidents.	
Social connections	PARO [41-46] and other social robots [47-49]	A sensor-based therapeutic robot that could improve people with dementia’s mood, social interaction, and wellbeing, which could be further improved via 6G and AI technologies: Connect with more advanced AI health surveillance systems. Computer vision for assistive medical diagnosis based on facial images [50], which could further facilitate personalized care design and development. Video-based vital signs monitoring (eg, Oxehealth [51]). Brain-machine interface devices, like Neurable [52], to gain insights into patients’ focus and productivity using headphones outfitted with EEG sensors. Enable assistive robots with more competent health monitoring and managing functions. Connect patients with their loved ones remotely “through” robots (eg, telepresence robots, which can transit voices, mimics, and head motions [53]). Transform assistive robots into multi-functional care assistants (eg, perform memory evaluation test [54]; provide dementia patients with a wider range of services, from fetch and carry, fall detection, to transfer patients from floor to chair/bed [55,56]).	

## Discussion

### Principal Findings

Our study aimed to investigate ways in which 6G and AI technologies could elevate dementia care. This is the first study that examined how society at large could better meet people with dementia’s care needs and wants with the aid of advanced technologies such as 6G and AI. From a technological perspective, by pointing out areas where 6G and AI could help further leverage dementia care, the study shed light on the importance of ingenuity and interoperability across the older (eg, 4G and 5G) and the newer networking platforms (eg, 6G) and analytical capabilities (eg, AI algorithms). From a care perspective, by detailing the promise and prowess of advanced technologies like 6G and AI in leveraging dementia care, the current study highlighted the importance of developing people-centric health solutions that could benefit the betterment of society and humanity, above and beyond the advancement of niche technologies. Overall, two fundamental research questions were answered to sufficiently address the research objective: (1) what are the unique characteristics of dementia care, and (2) what 6G and AI-powered technologies could address the unique needs and wants of dementia patients.

### Unique Characteristics of Dementia Care

While much remains to be known about dementia, meaningful understandings about people with dementia are available in the literature. Overall, a bevy of definitions of dementia was present in the literature, ranging from the ones delineated by national or international organizations (eg, World Health Organization, the United Kingdom’s National Health Service, the US Centers for Diseases Control and Prevention, the Government of Ireland, etc) [4,5,28-30], to conceptualizations developed to echo the unique characteristics of the research contexts [8,31-34]. Noticeably, almost all reviewed conceptualizations of dementia share the following similarities: (1) dementia is an abnormal part of aging that represents a diverse range of health conditions that are hallmarked by the gradual decline of (2) cognitive and (3) physical functionalities. In other words, though dementia is common among older people, it is not a condition of aging—young people can develop dementia, and some older people do not have dementia [5].

Furthermore, rather than one disease, dementia represents a family of cognitive impairments, which includes conditions such as Alzheimer’s Disease, vascular dementia, Lewy body dementia, and mixed dementia, often caused by varied disease manifestations [28-30]. To further complicate the situation, people can have multiple dementias at the same time or across the dementia continuum, conditions which are often difficult to detect or diagnose due to the similarities between types of dementia and limitations in medical sciences [28-30]. For instance, it is not uncommon for people with Alzheimer’s Disease to simultaneously have vascular dementia (linked to issues with blood flow to the brain), especially among older people.

Subsequently, from the perspective of dementia care, the unique characteristics of dementia symptoms could be further classified as disease manifestations that are related to cognitive impairments and the ones rooted in the decline of physical functions. Overall regardless of disease stage, from the patient’s perspective, across the dementia continuum, changes they experience regarding their dementia condition are often shown as fluctuations in cognitive and/or physical capabilities. In other words, people with dementia who experience optimal cognitive and physical abilities may have little to no need to rely on other people or advanced technologies to assist with their activities, whereas for individuals who experience poor cognitive and physical functionalities (eg, have difficulties in remembering, thinking, reasoning, walking, or moving), exterior help like advanced technologies are have-to-have enablers of their self-care needs and wants. A detailed illustration of the interplay between people with dementia’s abilities and their need for health technologies can be found in Table 3.

**Table 3:** The interplay between people with dementia’s abilities and their need for health technologies.

	High cognitive abilities	Low cognitive abilities
High physical abilities	Little to no dependence on assistive technologies. Health technologies are nice-to-have add-ons to daily activities.	Extremely high dependence on assistive technologies. Health technologies are have-to-have directors of daily activities.
Low physical abilities	High dependence on assistive technologies. Health technologies are have-to-have assistants of daily activities.	Extremely high dependence on assistive technologies. Health technologies are have-to-have enablers of daily activities.

### 6G and AI-Powered Technologies for Dementia Care

Compared to previous networking technologies such as 5G, 6G is considerably advanced, ranging from high-capacity connectivity to powerful real-time data analytics [57-64]. Overall, the relationship between 6G and AI will be that of symbiotic [65]—6G will lay the groundwork needed for the ecosystem to exist, whereas AI will make the 6G-enabled infrastructure produce meaningful health solutions for patients in a timely fashion, which in turn, facilitate the intelligentization of the hyper-connected community of solutions for dementia care. It is worth noting that though not all technical details of 6G have been fully explored, available research on its advantages in the following aspects renders investigations into 6G and AI-empowered dementia care justifiably relevant and urgent [65-68]: (1) 6G will address the technical limitations of previous networks (eg, 5G) with markedly superior communication solutions, (2) 6G can help elevate and enrich AI applications’ full potentials, (3) 6G technologies coupled with AI techniques can truly materialize smart dementia care—networking infrastructure with the ability to seamlessly transform data points into evidence-based and intelligent decision-making processes [69], and in turn, (4) offer much-needed insights into dementia care and management from a big data analysis perspective, effectively bridging some of the most ingrained research gaps in the literature (eg, lack of large population-based empirical research) [47].

For instance, Project Relate is an AI-powered communication tool developed by Google that aims to help people with speech impairments communicate smoothly [37]. Different from the general public, people with speech impairments, such as dementia patients, often exhibit more obtuse or convoluted linguistic characteristics [70]. By first asking people to record their personal speech and then analyzing and identifying the unique linguistic identifiers using AI-powered algorithms, Project Relate can subsequently help users “translate” what could be considered difficult-to-understand speeches into comprehensible conversations [37]. In other words, by building their own virtual villages with personal data, advanced technological solutions such as Project Relate could help dementia patients with speech impairments communicate with others more effectively and smoothly. The algorithms and the logics behind Project Relate could have revolutionary impacts on existing dementia care solutions that patients have full control over, such as social robots.

Social robots are interactive robots that could offer companionship and serve as an emotional or mental health booster for dementia patients [71-73]. In a study on the effects of PARO, a robotic seal, for instance, researchers found that the social robot helped dementia patients uphold their sense of life, increased their perceptions of social connection, and improved their positive emotions [74]. However, the use and application of social robots in dementia care face a wide range of issues [75,76]. For instance, the way PARO works is that it has a tactile stimulation-enabled “value” system, which dictates its rule of engagement: the robotic seal enjoys being stroked and dislikes being hit [72]. In its relationship with its owner, PARO is programmed to remember if it’s been stroked and act in ways that make it more likely to be stroked—subsequently, it gradually learns to develop a “personality” that its owner might appreciate [72]. In other words, when it comes to interacting with dementia patients, PARO’s technical capabilities allow it to be reactive and receptive but fall short of equipping it with capabilities to be proactive and responsive. Not to mention that one of the most noticeable flaws of social robots is that they cannot initiate verbal communication with dementia patients.

One way to address this issue is via 6G and AI technologies. For instance, when pair advanced AI algorithms such as those applied in health solutions like Project Relate with real-time data analytics enabled by 6G technologies, social robots will no longer serve as reactive companions to dementia patients but function as proactive caregivers that can provide intelligent health analytics (eg, noninvasive video-based health monitoring systems like Oxehealth) [51] and decision assistance to patients. In other words, as indicated in Table 2, social robots enhanced with 6G and AI technologies could have the potential to: (1) detect and predict patients’ agitation and/or violence episodes prior to disease onset or progression, (2) initiate communications, rather than waiting to be approached, (3) engage the patients with a wide range of interactions (eg, speech, physical interactions, etc) prior to the onset of aggression or violence, rather than solely based on reactive motion-activation technologies, and (4) process and analyze data gained from the interactions to build a patient-by-patient database system to facilitate personalized dementia care.

### Advanced Technology-Enabled Work

**Figure 2:**
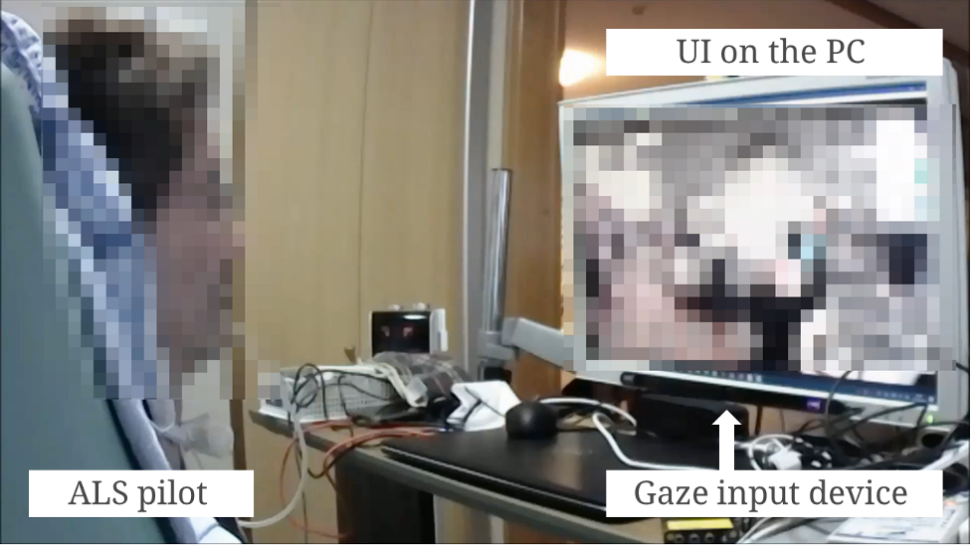
User interface of the “Avatar robot café” AI system.

**Figure 3:**
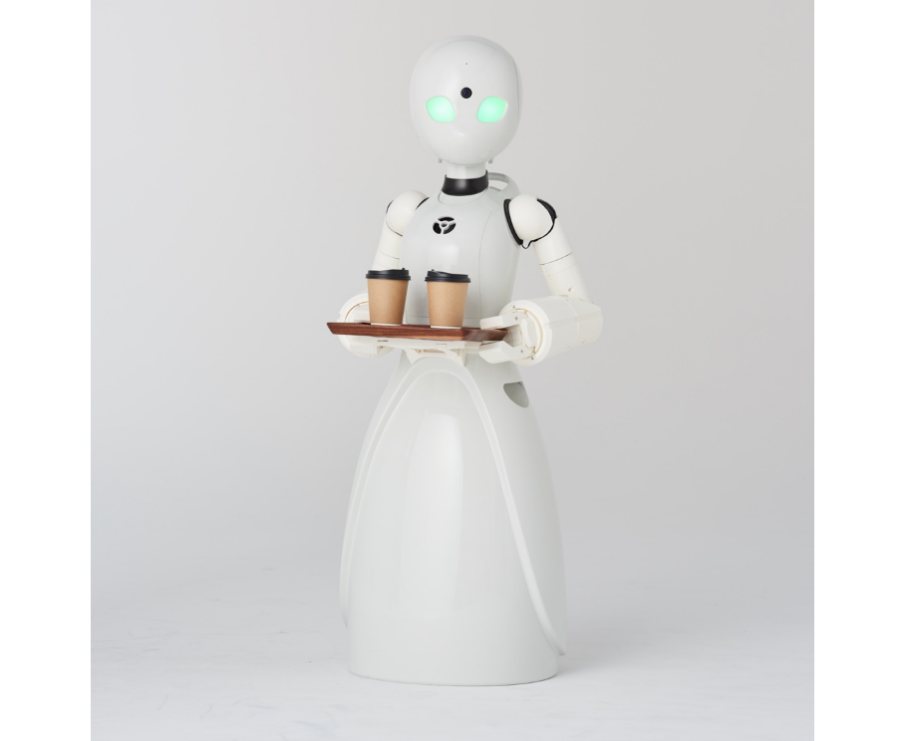
An example of piloted robot in action.

Recent advancements, such as the “avatar work” model, could further expand the promises of advanced technology-enabled dementia care [39,77]. With the help of advanced AI technologies, researchers are able to build systems that could enable people with physical impairments to “work” as robots in cafés that are appropriately named as Diverse Avatar Working Network (DWAN) [78]. Essentially, what is innovative about the “avatar work” or the “Work-Remotely-Café” model is that it allows people who are house-bound or bed-ridden, such as dementia patients, to work remotely in the form of physical robots via virtually controlling these robots using an AI-powered system at home or even in bed (Figure 2) [39]. In other words, what the DAWN model shows is that, with the help of advanced AI-powered algorithms, people with dementia could work as pilots of the robots (Figure 3).

It is important to underscore that even people with severe debilitating physical impairment can benefit from the AI-powered robots—similar to the abovementioned Project Relate, only minimum motor capabilities are required for people to work as pilots. Unfortunately, at the moment, the DAWN avatar robot café is only available in Japan, and its first permanent location opened in June 2021 in Tokyo [39]. However, in light of the fast development of AI technologies and the ever-expanding dementia population [79-81], it is safe to assume there will be similar or more advanced 6G and AI-powered frameworks that would enable “limited-mobility-yet-unlimited-opportunities” for people with dementia in the future.

### Limitations

While this study bridges vital gaps in the literature, it is not without limitations. First, the current study only focused on care solutions for people with dementia rather than informal caregivers or formal caregivers like health care professionals. To address this limitation, future research could investigate dementia health solutions that are designed for stakeholders other than the patients. Furthermore, in light of the parameters of our research aim, we only gauged advanced technology-based dementia care solutions in the context of 6G and AI opportunities. It is important to note that nontechnology-based dementia care solutions as well as technologies that are beyond the scale and scope of 6G and AI also play a critical role in dementia care [82,83]. In addition, the current study is not a systematic review, a limitation that could be sufficiently addressed as evidence continues to accumulate.

### Conclusions

To live is to grow old. Yet dementia is neither a proper way to live nor a natural way to grow old. While scientists continue to work on finding effective pharmaceutical interventions that could help cure or cub dementia, speedy, supportive, and successful non-pharmaceutical interventions are needed to address and alleviate the everyday health challenges people with dementia face. By identifying advanced health solutions powered by 6G and AI opportunities, our study shed light on how patients with dementia can live and prosper.

## Abbreviations

6G: sixth-generation communication solutions
AI: artificial intelligence
DWAN: Diverse Avatar Working Network

## Data Availability

Data are available upon reasonable request.
